# The different prognostic significance of polysialic acid and CD56 expression in tumor cells and lymphocytes identified in breast cancer

**DOI:** 10.1038/s41523-022-00442-w

**Published:** 2022-07-02

**Authors:** Sepideh Soukhtehzari, Richard B. Berish, Ladan Fazli, Peter H. Watson, Karla C. Williams

**Affiliations:** 1 Faculty of Pharmaceutical Sciences, The University of British Columbia, Vancouver, BC V6T 1Z3 Canada; 2 Vancouver General Hospital and Department of Urologic Sciences, The University of British Columbia, Vancouver, V6H 3Z6 BC Canada; 3 Deeley Research Centre, BC Cancer Agency, Vancouver Island Centre, University of British Columbia, 2410 Lee Avenue, Victoria, BC V8R 6V5 Canada

**Keywords:** Tumour biomarkers, Prognostic markers, Breast cancer, Cancer microenvironment

## Abstract

Protein glycosylation, the attachment of carbohydrates onto proteins, is a fundamental process that alters the biological activity of proteins. Changes to glycosylation states are associated with many forms of cancer including breast cancer. Through immunohistological analysis of breast cancer patient tumors, we have discovered the expression of an atypical glycan—polysialic acid (polySia)—in breast cancer. Notably, we have identified polySia expression in not only tumor cells but also on tumor-infiltrating lymphocytes (TILs) and our study reveals ST8Sia4 as the predominant polysialyltransferase expressed. Evaluation of ST8Sia4 expression in tumor cells identified an association between high expression levels and poor patient outcomes whereas ST8Sia4 expression in infiltrating stromal cells was associated with good patient outcomes. Investigation into CD56, a protein known to be polysialylated, found CD56 and polySia expression on breast tumor cells and TILs. CD56 expression did not positively correlate with polySia expression except in patient tumors which expressed HER2. In these HER2 expressing tumors, CD56 expression was significantly associated with HER2 expression score. Evaluation of CD56 tumor cell expression identified a significant association between CD56 expression and poor patient outcomes. By contrast, CD56 expression on TILs was significantly associated with good clinical outcomes. Tumors with CD56+ TILs were also consistently polySia TIL positive. Interestingly, in tumors where TILs were CD56 low-to-negative, a polySia+ lymphocyte population was still identified and the presence of these lymphocytes was a poor prognostic indicator. Overall, this study provides the first detailed report of polySia and CD56 in breast cancer and demonstrates that the prognostic significance is dependent on the cell type expression within the tumor.

## Introduction

Glycans are an essential part of all living cells, highlighted by the estimate that half of all human proteins are glycosylated. It is well recognized that glycans have major structural roles and mediate an extensive array of intrinsic and extrinsic actions in biological systems^1^. The generation of glycans through cellular glycosylation is a tightly controlled process involving the coordinated actions of specialized enzymes such as glycosyltransferases. Changes to protein glycosylation states are associated with several diseases and altered glycosylation is a notable trait in many forms of cancer including breast cancer^2–7^. There is clear significance for glycans in normal physiology and altered expression in cancer. The unveiling of alterations to the glycan content of cancer, including breast cancer, is a rapidly advancing and evolving field with great potential for therapeutic application^5,8,9^.

Glycosylation changes associated with oncogenic transformation of cells can arise through transcriptional and metabolic reprogramming resulting in the neosynthesis of specific glycoepitopes. Changes in the glycan composition of a neoplastic cell are associated with multifarious changes in cancer from promoting tumor proliferation to metastasis and immune evasion^4,10,11^. Increased sialylation is a notable trait of many cancer types and promotes tumor progression and, in breast cancer, elevated sialylation promotes tumor progression^12,13^. Polysialylation is a selective and highly regulated glycosylation event generating long glycan chains composed of α2,8-linked sialic acid residues, termed polysialic acid (polySia). Synthesis of polySia is performed by Golgi-localized sialyltransferase ST8Sia2 or ST8Sia4^14^. In healthy adults, polySia expression is restricted to a limited number of cell types and protein carriers^15^. Classically, polySia has been documented on neuronal cells, but it is now recognized that polySia also occurs in the context of the immune system. PolySia has been found on both human and murine leukocytes^16^. Specifically, in human leukocytes polySia has been found on natural killer (NK) cells and CD3+ T cells^16,17^. CD56 (also known as neural cell adhesion molecule, NCAM-1) was identified as a carrier protein for polySia chains on NK and CD3+ T cells. In the context of cancer, neoexpression of polySia has been documented in multiple cancers, such as neuroblastoma, glioma, lung carcinoma, and leukemia^15,18^. Overexpression of polySia in cancer cells is suggested to increase migration and invasion, and in clinical biopsy specimens from glioblastoma and non-small cell lung cancer (NSCLC) polySia expression strongly correlated with metastasis and poor prognosis^19–21^. CD56 was identified as the carrier protein of polySia chains in glioblastoma. Interestingly however, in NSCLC, polySia-positive tumors were not always CD56 positive suggesting the presence of additional polysialylated proteins in cancer. PolySia has clear links to cancer progression and was ranked as the second highest priority glycan for investigation in cancer by a National Cancer Institute pilot project^22^. Here, we set out to examine the profile of expression and clinical relevance of polySia, together with CD56, and polysialyltransferases ST8Sia4 and ST8Sia2, in primary breast tumors.

## Results

### Polysialic acid expression in primary breast tumors

**Fig. 1:**
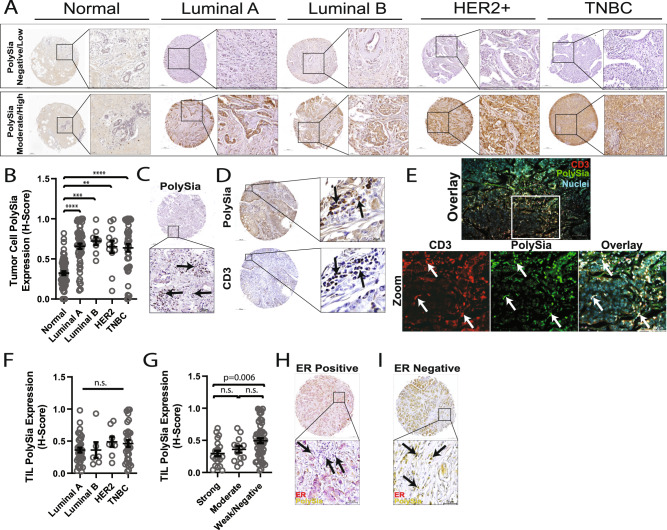
Polysialic acid expression in primary breast tumors. **A** Immunohistochemical staining of polysialic acid in normal tissue and breast cancer tissue. Representative images of normal breast tissue and tumors showing within each tissue or tumor category of low expression (top row) and high expression (bottom row). Cores are shown alongside higher magnification insets from the same core. **B** Analysis of polysialic acid expression (*H*-Score) in normal breast tissue and tumor cells categorized into molecular subtype. Luminal A (*n* = 60), Luminal B (*n* = 9), HER2+ (*n* = 12), and triple-negative breast cancer (TNBC) (*n* = 42). Bars indicate medians and ±SEM, ****P < 0.0001, Kruskal–Wallis test. Identification of polysialic positive tumor infiltrating lymphocytes in the tumor microenvironment by immunohistochemical staining of polysialic acid (**C**) and CD3 (**D**). Representative images are shown alongside higher magnification insets from the same core. Arrows point to lymphocytes positive for polySia (**C**) and regions of lymphocytes positive for both polySia and CD3 (**D**). **E** CD3 positive tumor-infiltrating lymphocytes express polySia. Breast tumor tissue was immunostained for CD3 (red) and polySia (green), followed by a nuclei stain (blue). Bottom row represents zooms from corresponding image in the top row. Arrows point to CD3-positive cells (red) which are also positive for polySia (green), and can be visualized as yellow in the overlay. Scale bar = 100 µm. **F** and **G** Analysis of polysialic acid expression (*H*-Score) on tumor-infiltrating lymphocytes categorized by molecular subtype (Luminal A, *n* = 36; Luminal B, *n* = 6; HER2+, *n* = 8; TNBC, *n* = 31) (**F**) or estrogen receptor expression (strong, *n* = 21; weak, *n* = 51; moderate:n = 13) (**G**). Representative images of polySia TIL expression in ER positive (**H**; arrows points to lymphocytes with low/no polySia) and ER negative (**I**; arrows point to yellow/polySia positive lymphocytes). Bars indicate medians and ±SEM, *p* values shown from Tukey’s test. Scale bar = 200 µm (cores) and 50 or 20 µm (insets).

To explore polySia expression in breast cancer, immunohistochemistry was performed on normal and adjacent normal breast tissue and primary breast cancer tumors. PolySia expression was evaluated using one TMA containing *n* = 65 healthy/normal breast tissue samples and two TMAs containing primary breast cancer tumors. Tumors were grouped into molecular subtypes based on the expression of ER, PR and HER2 (see Supplemental Fig. 1 for breakdown of all breast cancer TMAs, tissue sections, associated clinical data, corresponding experiments used, and molecular subtype grouping). The two breast cancer TMAs contained a total of *n* = 144 cases from primary breast tumors from which *n* = 123 contained data on all three (ER, PR, and HER2) receptor expression levels. These 123 cases were categorized further based on receptor expression as Luminal A (*n* = 60), Luminal B (*n* = 9), HER2+ (*n* = 12), and triple-negative breast cancer (TNBC) (*n* = 42). PolySia expression levels were evaluated for each molecular subtype (*n* = 123) and healthy/adjacent normal breast tissue (*n* = 65). In normal breast tissue polySia expression was low whereas polySia expression was significantly increased in all molecular subtypes of breast cancer (Fig. 1A and B). No significant difference in polySia expression was found between subtypes and all subtypes exhibited a wide-range of polySia expression from low expression to high expression (Fig. 1A and B). Further analysis based on individual receptor expression (ER, PR, HER2) did not identify any significant differences between polySia levels and receptor expression (Supplementary Data Fig. 2A–C). Interestingly, we also noted polySia expression on tumor infiltrating lymphocytes (TILs) (Fig. 1C). CD3 and polySia immunostaining of serial sections found that cells expressing polySia and CD3 colocalized in the same regions of the tumor microenvironment (Fig. 1D). To further confirm polySia expression by CD3+ lymphocytes dual fluorescence immunostaining was performed which confirmed that CD3+ lymphocytes were positive for polySia (Fig. 1E). Analysis of polySia expression levels on TILs relative to molecular subtype (Luminal A, *n* = 36; Luminal B, *n* = 6; HER2+, *n* = 8; TNBC, *n* = 31) demonstrated a trend towards increased polySia TIL levels in HER2+ and TNBC but this was not found to be significant (Fig. 1F). Further analysis of TIL polySia expression identified significantly higher levels of polysialylated TILs in ER weak/negative tumors compared to tumors with strong ER expression (Fig. 1G, H and I). No association was found with HER2 or PR expression (Supplementary Data Fig. 2D and E). PolySia expression on tumor cells and TILs did not significantly associate with tumor Grade, Stage, or patient age (Supplementary Data Fig. 2F–K). PolySia antibody specificity was validated through western blotting and immunohistochemistry on cell line generated tumor xenografts (Supplementary Data Fig. 3). Overall, these results demonstrate that polySia is expressed in breast tumors and present on tumor cells and also tumor-infiltrating lymphocytes.

### Sialyltransferase, ST8Sia4 and ST8Sia2, expression in primary breast tumors

**Fig. 2:**
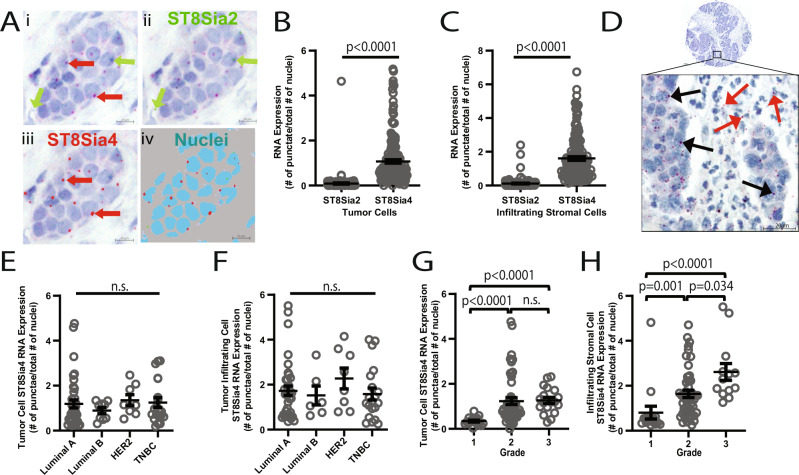
ST8Sia2 and ST8Sia4 expression in primary breast tumors. **A** ST8Sia4 and ST8Sia2 in situ hybridization (ISH) and identification of ST8Sia2 (ii), ST8Sia4 (iii), and nuclei (iv) using Intellesis software. Red arrows point to (i) ST8Sia4 RNA punctae from ISH staining and (iii) ST8Sia4 RNA punctae as identified by Intellesis software. Green arrows point to (i) ST8Sia2 RNA punctae from ISH staining and (ii) ST8Sia2 RNA punctae as identified by Intellesis software. Scale bar = 10 µm. **B** and **C** Quantification of the total number of ST8Sia2 and ST8Sia4 RNA punctae as a ratio of total number of tumor cell nuclei (**B**) or infiltrating stromal cell nuclei (**C**). ±SEM, unpaired *t* test with Welch’s correction. **D** Representative image of a ST8Sia4 high expressing tumor. Black arrows point to ST8Sia4 RNA punctae in tumor cells and red arrows point to ST8Sia4 RNA punctae in infiltrating stromal cells. Scale bar = 20 µm. **E** and **F** ST8Sia4 expression in (**E**) tumor cells (luminal A, *n* = 39; Luminal B, *n* = 9; HER2+, *n* = 8; TNBC, *n* = 20) and **F** infiltrating stromal cells (Luminal A, *n* = 37; Luminal B, *n* = 6; HER2+, *n* = 8; TNBC, *n* = 20) grouped by molecular subtype. **G** and **H** ST8Sia4 expression in **G** tumor cells (Grade 1, *n* = 17; Grade 2, *n* = 51, Grade 3, *n* = 18) and **H** infiltrating stromal cells (Grade 1, *n* = 16; Grade 2, *n* = 48, Grade 3, *n* = 13) grouped by overall tumor grade. ±SEM, Kruskal–Wallis test.

PolySia is exclusively synthesized by either sialyltransferase (ST) 8Sia4 or 2 (ST8Sia4 and ST8Sia2). To evaluate polysialyltransferase expression levels in breast tumor cells and TILs, in situ hybridization (ISH) was performed on tumor tissue cores and probed for ST8Sia2 and ST8Sia4 (Fig. 2A). Cores were evaluated for ST8Sia4 and ST8Sia2 expression in tumor cells and in infiltrating stromal cells. For both tumor cells and infiltrating stromal cells, ST8Sia4 was the predominant polysialyltransferase expressed whereas ST8Sia2 expression was significantly lower in tumor cells and infiltrating stromal cells relative to ST8Sia4 (Fig. 2B–D). ST8Sia4 expression in tumor cells and infiltrating stromal cells was not found to associate with any molecular subtype (Fig. 2E and F). ST8Sia4 tumor cell expression was found to significantly associate with a higher tumor Grade (Fig. 2G). Grade 1 tumors had significantly lower ST8Sia4 expression levels relative to Grades 2 and 3 tumors. Similar results were also found for infiltrating stromal cells as increased ST8Sia4 expression significantly associated with a higher tumor Grade (Fig. 2H). No significant differences were identified between ST8Sia4 expression and Stage and no significant correlation was found with age (Supplementary Data Fig. 4). ST8Sia4 RNA probe was validated using cell line generated tumor xenografts (Supplementary Data Fig. 5).

**Fig. 3:**
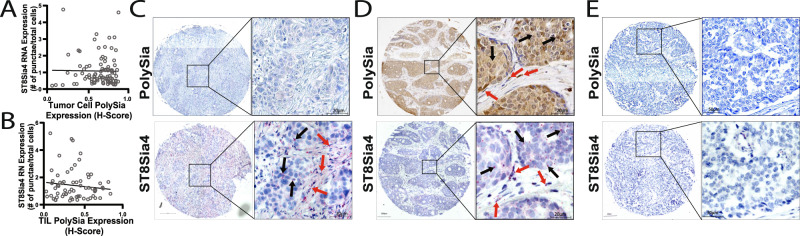
PolySia and ST8Sia4 expression in tumor cells and tumor-infiltrating lymphocytes. **A** and **B** Correlation between tumor cell (*n* = 93) (**A**) and (**B**) TIL (*n* = 70) polySia expression levels plotted relative to ST8Sia4 expression levels for each matched tissue core. Gray line = Regression line. Pearson correlation test and simple linear regression test. **C–E** Representative images of polySia and ST8Sia4 expression serial sections. Zooms are of similar regions for each matched set. Arrows point to tumor cells (black arrows) and lymphocytes (red arrows) positive for polySia and/or ST8Sia4. Scale bar = 200 µm (cores) and 50 µm (insets).

Next, to evaluate if ST8Sia4 expression correlates with polySia expression, we plotted total polySia expression relative to ST8Sia4 expression for each matched tumor core. No significant correlation between ST8Sia4 expression and polySia expression was identified for tumor cells (Fig. 3A; *n* = 93) and TILs (Fig. 3B; *n* = 70). We found that some tumors had high ST8Sia4 expression but lacked polySia expression (Fig. 3C) whereas other tumors displayed both ST8Sia4 expression and polySia expression (Fig. 3D). A representative image for a ST8Sia4 and polySia low/negative tumor is also shown (Fig. 3E). Potentially, the lack of a correlation between ST8Sia4 and polySia expression is a result of RNA expression not reflecting protein expression, or potentially it may be related to the presence or absence of a polySia carrier protein(s).

### Prognostic impact of ST8Sia4 and polysia expression in breast tumor cells

**Fig. 4:**
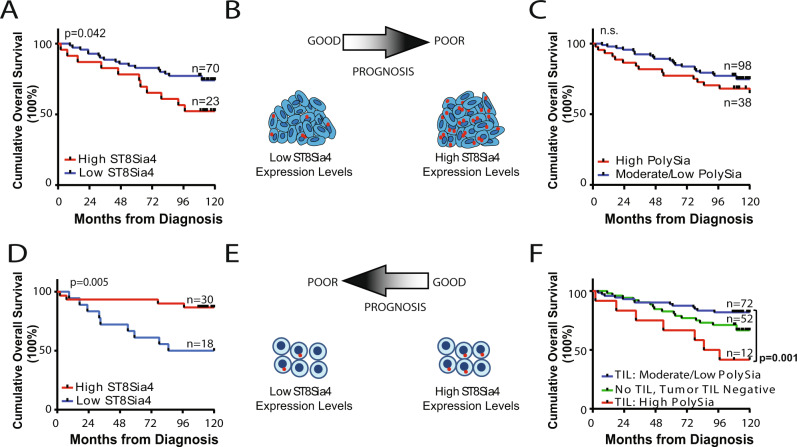
Kaplan–Meier curves demonstrating survival rates relative to ST8Sia4 and polysialic acid expression in tumor cells and tumor-infiltrating lymphocytes. **A** Overall survival of individuals based on ST8Sia4 expression in tumor cells. Patients were categorized as either ST8Sia4 high (>1.25 RNA punctae/cell) or low ST8Sia4 (≤1.25 RNA puncate/cell). **B** Animated diagram depicting the prognostic significance of ST8Sia4 expression in tumor cells. **C** Overall survival of individuals based on polysialic acid levels in tumor cells. Patients were categorized as high expression (*H*-Score > 0.66) or moderate/low (*H*-Score ≤ 0.66). High ST8Sia4 and polySia cut-point value was set at the 75% Percentile ±SEM of all values. Overall Survival curve comparison: Log-rank (Mantel–Cox) test. **D** Overall survival of individuals based on infiltrating stromal cell ST8Sia4 expression in TIL-positive tumor cores. Patients were categorized as either ST8Sia4 high (>0.4 RNA punctae/cell) or low ST8Sia4 (≤0.4 RNA punctae/cell). High ST8Sia4 cut-point value was set at the median ± SEM of all values. **E** Animated diagram depicting the prognostic significance of ST8Sia4 expression in infiltrating stromal cells. **F** Overall survival of individuals based on polysialic acid levels on TIL. Patients were categorized as high expression (*H*-Score > 0.55) or moderate/low (*H*-Score ≤ 0.55). TIL Negative (tumor cores absent for TIL). High polySia cut-point value was set at the 75% Percentile ±SEM of all values. Overall Survival curve comparison: Log-rank (Mantel–Cox) test.

To determine the prognostic potential of ST8Sia4 and polySia in breast tumor cells, ST8Sia4 and polySia expression levels were evaluated and correlated to patient overall survival in a cohort of invasive ductal carcinoma (IDC) cases with 10 years overall survival data (*n* = 136). Consecutive tissue microarray (TMA) sections were analyzed for polySia and ST8Sia4 expression. For ST8Sia4 analysis *n* = 93/136 tumor cores were amenable to analysis (exclusion was based on core loss and tissue deformation which prevented Intellesis analysis). Elevated ST8Sia4 expression in tumor cells was significantly associated with poor overall survival compared to tumors with low expression of ST8Sia4 (*p* = 0.04; HR [High/Low] = 2.47, 95% CI: 1.03–5.95; High [*n* = 23], Low [*n* = 70]) (Fig. 4A and B). Evaluation of polySia levels in tumor cells found no significant difference in survival outcomes for individuals with moderate to low polySia expression compared to individual with high expression (Fig. 4C). While polySia expression levels did not significantly correlate with patient outcomes over a 10-year period (*p* = 0.13; HR = 1.74, 95% CI: 0.85–3.60), we did note that individual with high polySia levels had a trend to worse outcomes, particularly within the initial 5-year period (*p* = 0.060) (5-year HR = 2.56, 95% CI: 0.96–6.42).

### Prognostic impact of ST8Sia4 and PolySia expression in tumor-infiltrating lymphocytes

Next, we sought to evaluate the prognostic significance of ST8Sia4 and polySia expression in TILs. Breast tumor cores used to evaluate tumor cell ST8Sia4 and polySia levels, were analyzed for ST8Sia4 expression and polySia expression levels in TILs. Consecutive TMA sections were analyzed for polySia and ST8Sia4 RNA expression. Analysis of ST8Sia4 expression in TIL-positive tumors (*n* = 84) was limited to 48 cores (exclusion was based on core loss, tissue damage, or tissue deformation which prevented Intellesis analysis) and revealed that high levels of ST8Sia4-expressing cells in the stroma were significantly associated with better patient outcomes (Fig. 4D). Individuals with low levels of ST8Sia4 expressing stromal cells had significantly worse outcomes compared to individuals with high ST8Sia4 expression (*p* = 0.005; HR [Low/High] = 5.25, 95% CI: 1.64–16.8; High [*n* = 30], Low [*n* = 18]). This indicates that the elevated levels of ST8Sia4 positive-infiltrating stromal cells identifies individuals with good long-term, 10 year, outcomes (Fig. 4E).

Analysis of polySia levels on TILs was also evaluated and correlated to patient outcomes in all 136 tumors (Fig. 4F). TILs were present in 84/136 tumors and in this subset high levels of polysialylated TILs were significantly associated with poor overall survival compared to moderate/low polysialylation which was a good prognostic indicator (*p* = 0.0016; HR [High/Moderate] = 8.7, 95% CI: 2.27–33.61; High [*n* = 12], Moderate/Low [*n* = 72]). Individuals with moderate/low polysialylated TIL had a trend towards improved overall survival compared to those with TIL-negative tumors (*n* = 52; tumors with no lymphocyte infiltration) (*p* = 0.07). These results are in discordance with our results demonstrating that ST8Sia4 expression was a favorable prognostic factor. Potentially this is a consequence of ST8Sia4 RNA expression not reflecting protein expression, or potentially it is related to the expression of the polySia carrier protein(s) as ST8Sia4 can only generate polySia chains in the presence of a polySia acceptor protein. In addition, as moderate polySia expression was favorable, our assessment of the extreme/high polySia expressers may reflect a unique population.

### Evaluation of polySia and CD56 expression in breast cancer

**Fig. 5:**
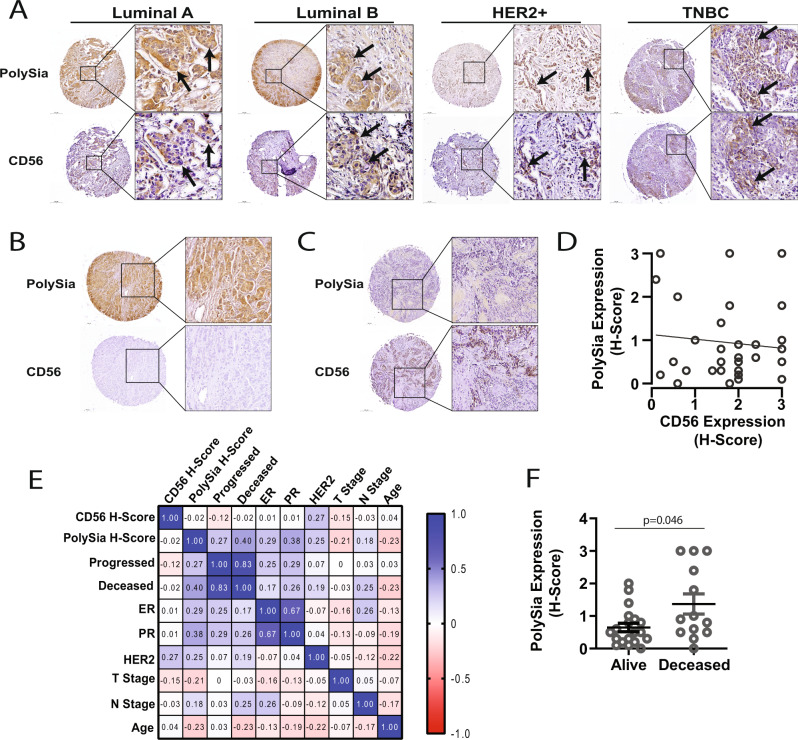
PolySia and CD56 expression in primary breast tumors. **A–C** Immunohistochemical staining of polySia (top row) and CD56 (bottom row) in each panel in breast cancer tissue. Representative images are shown alongside higher magnification insets from the same core. Arrows point to clusters of cells positive for polySia and CD56. Representative images are shown alongside higher magnification insets from the same core. Scale bar = 200 µm (cores), or 50 µm (insets). **D** Correlation between polySia expression levels plotted relative to CD56 expression levels for each matched tissue core (*n* = 32; gray line: regression line). Pearson correlation test and simple linear regression test. **E** Correlation matrix of all clinical data and polySia and CD56 expression (*n* = 32). Color map represents correlation strength between each data set; represented as positive (blue) or negative (red). Pearson *R* values are indicated in each box. **F** PolySia expression levels categorized based on disease specific patient outcome (Alive [*n* = 19], Deceased [*n* = 13]). ±SEM, unpaired *t*-test.

To evaluate polySia expression in the context of a polySia carrier protein we assessed CD56, also known as neural cell adhesion molecule (NCAM), expression in breast tumor tissue. CD56 antibody specificity was validated by western blot and IHC on tumor xenografts (Supplementary Fig. 6). A general examination of CD56 and polySia using adjacent serial breast tumor core sections found expression in each subtype (Fig. 5A). While CD56 was expressed by tumor cells in ~15% of tumors, ~60% of tumors were positive for polySia suggesting that other proteins are polysialylated in breast tumor cells (Fig. 5B). In addition, of the CD56-positive tumors only ~55% were polySia-positive suggesting that CD56 expression does not infer polySia expression (Fig. 5C). To further evaluate CD56 expression in breast cancer we collected a small cohort (*n* = 33) of breast cancer cases with whole tumor tissue embedded in paraffin blocks and clinical follow-up data (*n* = 33; Luminal A [*n* = 11], Luminal B [*n* = 3], HER2 [*n* = 6], TNBC [*n* = 13]). Whole tumor tissue sections were immunostained for CD56 and polySia (serial sections) and expression levels of CD56 and polySia were analyzed relative to multiple variables: tumor staging, receptor expression, disease progression/recurrence and disease-specific overall survival. An initial evaluation of the correlation between polySia levels and CD56 expression in this cohort was not found to be significant (Pearson *R* = −0.1091) (Fig. 5D). Next, we evaluated polySia and CD56 expression relative to all clinical parameters and patient outcomes using a Correlation matrix of all clinical data (Fig. 5E). Patients, to date, had a minimum of 3 years clinical follow-up documenting disease-specific recurrence and disease-specific overall survival status. One patient was lost to follow-up at 14 months and was excluded from the overall survival analysis. Results from the Correlation matrix analysis found, as expected, that clinical parameters such as progression status, ‘Progressed’, and *N* stage were positively correlated with a status of ‘Deceased’ (Fig. 5E). PolySia expression was found to positively correlate with patients whose disease progressed (Pearson *R* = 0.273) and disease-specific death (Pearson *R* = 0.402). In this small cohort, polySia expression was found to be significantly higher in patients who died from their disease (*p* = 0.046) (Fig. 5F; Alive [*n* = 19], Deceased [*n* = 13]). CD56 did not significantly correlate with any clinical parameter or polySia expression but a positive correlation was found for CD56 expression and HER2 status (Pearson *R* = 0.271) (Fig. 5E). Taken together this data supports the presence of polySia and CD56 expression in breast cancer and identifies a potential association between CD56 and HER2 expression.

### Prognostic impact of polySia and CD56 expression in HER2+ breast cancer

**Fig. 6:**
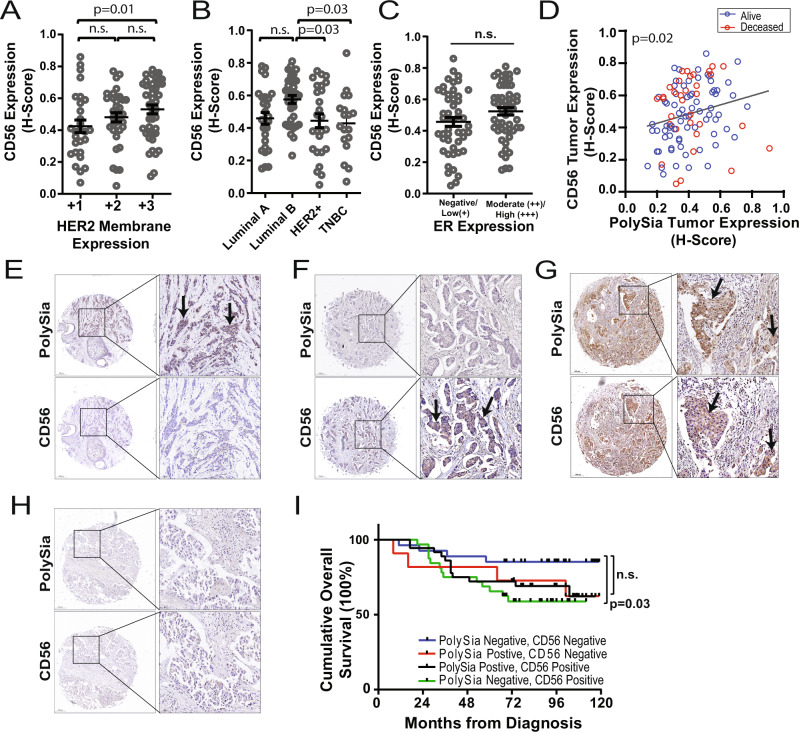
PolySia and CD56 tumor cell expression in HER2 expressing tumors and correlation with patient outcomes. **A–C** Analysis of CD56 expression (*H*-Score) in HER2 expressing tumors (*n* = 106) categorized by **A** HER2 membrane expression levels, **B** molecular subtype, or **C** ER expression. Bars indicate medians and ±SEM, *p* < 0.05 denotes significance. Tukey’s Test (**A**, **B**) and unpaired *t*-test (**C**). **D** Correlation between CD56 expression levels plotted relative to polySia expression levels for each matched tissue core. Each data point was pseudo-colored based on patient outcome (Red = Deceased, Blue = Alive). Gray Line = Regression line for all matched tissue cores. Pearson correlation test and simple linear regression test. **E–H** Immunohistochemical staining of polySia (top row) and CD56 (bottom row) in each panel in breast cancer tissue. Representative images are shown alongside higher magnification insets from the same core. Arrows point to clusters of cells positive for polySia and/or CD56. Representative images are shown alongside higher magnification insets from the same core. Scale bar = 200 µm (cores), or 50 µm (insets). **I** Kaplan–Meier curves for overall survival of individuals based on polySia and CD56 expression levels in tumor cells. Patient tumors were categorized as polySia-CD56 positive (*n* = 36), CD56 positive/polySia negative (*n* = 32), polySia positive/CD56 negative (*n* = 11) and polySia low/negative, CD56 low/negative (*n* = 27). Tumors with a *H*-Score > 0.4 for each marker were classified as polySia and/or CD56 positive; cut-point value was set at the median ± SEM of all values. Overall survival curve comparison: Log-rank (Mantel–Cox) test.

To further assess the correlation between CD56 expression and HER2 expression we obtained a TMA containing HER2-expressing breast tumors. The TMA contained *n* = 106 HER2-expressing IDC breast tumor cores with 5–10 years clinical follow-up data reporting on patient outcomes. Adjacent serial sections were immunostained for CD56 and polySia. Analysis of CD56 expression based on HER2 expression (+1, low; +2, moderate; +3, strong) demonstrated that CD56 expression increased with HER2 receptor expression and CD56 expression was significantly higher in HER2 positive (3+) tumors compared to HER2 negative/low (1+) tumors (Fig. 6A; *p* = 0.01, *n* = 106). Evaluation of CD56 expression levels relative to molecular subtype (*n* = 106: Luminal A, *n* = 28; Luminal B, *n* = 35 HER2+, *n* = 25; TNBC, *n* = 18) identified a significant association with Luminal B tumors (Fig. 6B), however no significant association was found with ER expression (Fig. 6C). Evaluation of polySia and CD56 expression in this HER2 expressing cohort identified a significant correlation between polySia and CD56 expression (Fig. 6D; Pearson *R* = 0.22 [gray regression line], *p* = 0.02). While the majority of tumors expressing polySia were also positive for CD56, we did note a small number of polySia positive, CD56 low/negative tumors. CD56 positive, polySia negative tumors were also identified, and consistent with our initial findings approximately half of the CD56-positive tumors (*H*-Score > 0.4) were polySia low/negative (*H*-Score < 0.4). To analyze expression relative to patient outcomes we grouped patients into four categories: (1) polySia positive, CD56 negative (*n* = 11) (Moderate or High polySia and Negative or Low CD56 expression; Fig. 6E), (2) polySia negative, CD56 positive (*n* = 36) (Negative or Low polySia, and High or Moderate CD56; Fig. 6F), (3) polySia and CD56 positive (*n* = 32) (High or Moderate polySia, and High or Moderate CD56; Fig. G), and (4) polySia and CD56 negative (*n* = 27) (Negative or Low expression; Fig. 6H). Analysis of patient outcome relative to polySia and CD56 expression identified a significant association between high levels of CD56 and poor patient outcomes (*p* = 0.03, HR [PolySia Negative, CD56 Positive/PolySia Negative, CD56 Negative] = 2.76, 95% CI: 1.06–7.09) (Fig. 6I). Expression of polySia and CD56 was also found to trend with worse patient outcomes, although this did not meet significance (*p* = 0.11, HR [polySia Positive, CD56 Positive/PolySia Negative, CD56 Negative] = 2.21, 95% CI: 0.82–5.95).

**Fig. 7:**
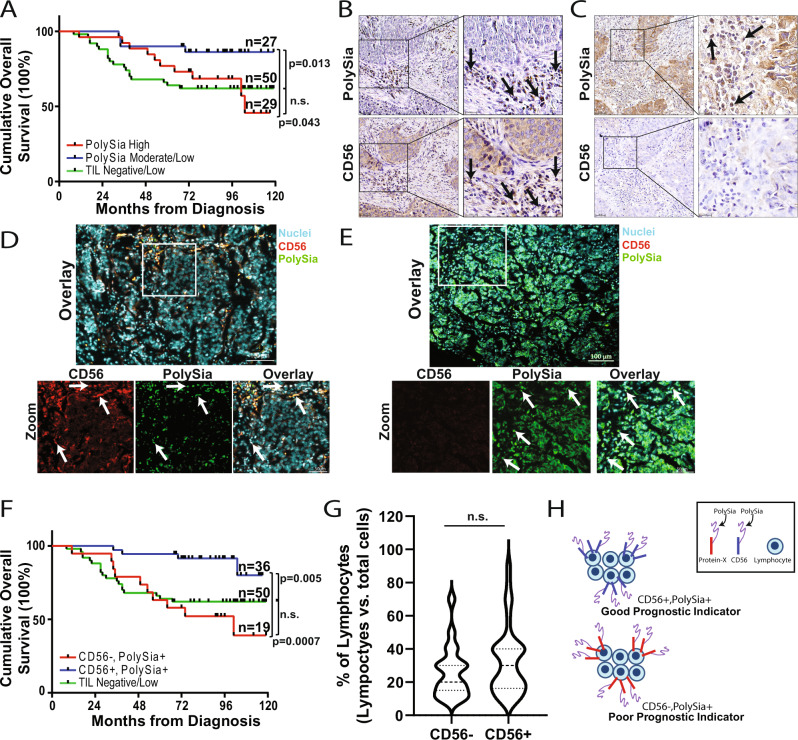
PolySia and CD56 lymphocyte expression in HER2 expressing tumors and correlation with patient outcomes. Kaplan–Meier curves for overall survival of individuals based on polySia expression levels in lymphocyte cells. Patient tumors were categorized as high polySia expression (*H*-Score ≥ 0.135) or moderate/low (*H*-Score ≤ 0.135). High polySia cut-point value was set at the 75% Percentile ±SEM of all values. **B** and **C** Immunohistochemical staining of polySia (top row) and CD56 (bottom row) in breast tumors categorized as TIL positive. Representative images are shown alongside higher magnification insets. Arrows point to regions of TILs positive for polySia and/or CD56. Scale bar = 50 and 20 µm (zoom). **D** and **E** Breast tumor tissue was immunostained for CD56 (red) and polySia (green), followed by a nuclei stain (blue). Bottom row represents zooms from corresponding image in the top row. **D** Arrows point to CD56 positive cells (red) which are also positive for polySia (green), and can be visualized as yellow in the overlay. **E** Arrows point to polySia cells which are negative for CD56. Scale bar = 100 or 50 µm (zoom). **F** Kaplan–Meier curves for overall survival of individuals based on polySia and CD56 TIL expression. Lymphocytes with a *H*-Score > 0.06 for each marker were classificed as polySia and CD56 postive; cut-point value was set at the 25% percentile ±SEM of all values. Overall Survival curve comparison: Log-rank (Mantel–Cox) test. **G** Percentage of lymphocytes in each tumor core categorized by TIL CD56 expression. **H** Animated diagram depicting the potential prognostic significance of polySia and CD56 expression in lymphocytes.

Of the 106 HER2 expressing tumor cores, 56 cores were positive for TILs (lymphocytes ≥10% of total cells/core). An evaluation of polySia and CD56 expression on TILs found that polySia and CD56-positive TILs were readily detectable in tumors. In line with our results (Fig. 4F), when we evaluated total polySia TIL levels relative to patient outcomes by separating out high polySia TIL-expressing tumors (*n* = 29) and comparing these to moderate/low polySia TIL-expressing tumors (*n* = 27), we found high polySia TIL expression to be a poor prognostic factor (Fig. 7A). Evaluation of CD56 TIL levels in serial tissue sections found that the majority of tumors containing CD56-positive TILs (*n* = 36) also contained polySia-positive TILs localized to the same regions (Fig. 7B). Only one tumor was negative for both polySia- and CD56-positive TILs. Interestingly, in the remaining 19 tumors which lacked CD56-expressing TILs we found that these tumors still contained polySia-positive TILs (Fig. 7C). To further support these findings, co-localization of polySia with CD56 was assessed using immunofluorescence staining which identified polySia+, CD56+ and polySia+, CD56− TILs (Fig. 7D and E). This suggests that polysialylated CD56 TILs are present in the tumor microenvironment and potential other, CD56 negative, TILs carry different polysialylated proteins.

Here we identified polySia+ TILs in tumors containing CD56+ TILs and in tumors lacking CD56+ TILs. As such, different populations of polySia-expressing lymphocytes could have distinct prognostic significance. To evaluate this, tumors were classified based on lymphocyte expression as either polySia+, CD56+ (Fig. 7B) or polySia+, CD56− (Fig. 7C). Of the *n* = 56 cores only one was low/negative for both CD56 and polySia and this core was excluded for the analysis. Individuals with tumors containing CD56+, polySia+ TILs (*n* = 36) had significantly better overall survival compared to individuals whose tumors contained CD56−, polySia+ TILs (*n* = 19) (*P* = 0.0007; HR [CD56−,PolySia+/CD56+,PolySia+] = 7.32, 95% CI: 2.32–23.0) (Fig. 7F). In addition, individuals with tumors containing CD56+/polySia+ TILs had significantly better survival outcome compared to individuals with TIL-negative tumors (*p* = 0.005, HR [TIL Low/CD56+, PolySia+] = 3.24, 95% CI: 1.42–7.40). Evaluation of the percentage of TILs per tumor core found no significant difference in TIL levels between tumors with CD56+ TILs and CD56− TILs (Fig. 7G). Overall, this demonstrates that the presence of polySia+, CD56+-expressing TILs in patient tumors is a good prognostic indicator. We also discovered the presence of polySia-positive lymphocytes in tumors lacking CD56 positive TILs and found that the presence of these lymphocytes is a poor prognostic indicator (Fig. 7H).

## Discussion

This study is, to our knowledge, the first comprehensive analysis of polySia, CD56, and polysialyltransferases ST8Sia2 and ST8Sia4, in primary breast tumors. This is also the first identification of polysialylated TILs in the tumor microenvironment. Our work details the prognostic significance of polySia, CD56, and ST8Sia4 in breast cancer adding to the current literature in support of a role for polySia in cancer progression. However, our findings also add complexity to the role of polySia in cancer and suggest that different polysialylated proteins and/or cell type expression may have distinct prognostic capabilities.

Our findings detail polySia and ST8Sia4 tumor cell expression in breast cancer. Neither polySia nor ST8Sia4 was associated with any molecular subtype. Indeed, all molecular subtypes exhibited varying expression levels of polySia and ST8Sia4. ST8Sia4 tumor cell expression was found to significantly associate with tumor grade and poor patient outcomes, and polySia tumor cell expression trended towards worse outcomes. We found that ST8Sia4 expression, as assessed by RNA ISH, did not correlate with polySia expression which could suggest that ST8Sia4 RNA expression does not reflect ST8Sia4 protein levels. Or, potentially, the lack of correlation could be due to the lack of expression of a polySia carrier protein(s) in some ST8Sia4-positive tumors. Our study did not assess ST8Sia4 protein expression primarily due to difficulties validating the specificity of ST8Sia4 antibodies. Another potential limitation of our study is the size of our cohorts. Analysis of additional clinical samples would have strengthened our work and improved our statistical output.

ST8Sia4 was found to be expressed by infiltrating stromal cells and the presence of these cells was found to be a good prognostic indicator. ST8Sia4 is known to be expressed by multiple immune cell subtypes including CD4+ T helper lymphocytes^23^, NK cells^16,17^ and macrophages^24^. As our analysis of ST8Sia4 included all cells in the tumor stromal space, it is possible that different populations of ST8Sia4-expressing stromal cells were present and each population(s) may have unique significance for patient outcomes. While ST8Sia4 expression was a favorable prognostic indicator, high levels of polySia expression on TILs was found to be a poor prognostic indicator relative to moderate/low polySia expression. The discordance of these results could potentially be a consequence of unique lymphocyte subpopulation(s) expressing different polysialylated proteins at varying levels, each with their own prognostic significance, or due to ST8Sia4 RNA levels not reflecting protein levels.

Next, we evaluated the expression of CD56, a well-known polySia carrier protein, in breast cancer. Here, we clearly show that CD56 is expressed in a subset of tumors and in approximately half of these tumors, tumor cells are also polySia positive. This strongly suggests the presence of polysialylated CD56 in breast tumor cells. Importantly, we also clearly identify polySia positive, CD56 negative tumor cells indicating that additional proteins are polysialylated in the context of breast cancer. Similar results were found for lymphocytes where polySia+ TILs were found to be CD56+ in some patient tumors, while other tumors had high levels of polySia+ TILs but lacked CD56. Further work is needed to identify the polySia protein carrier(s) in CD56-negative tumor cells and TILs. Potentially, different protein carriers and cell populations have distinct prognostic capacity.

Our evaluation of CD56 in breast cancer found a positive and significant association with HER2 expression. In addition, in this HER2-expressing cohort CD56 expression positively correlated with polySia expression. High levels of CD56 and polySia-CD56 expression on tumor cells was associated with poor patient outcomes. However, while polySia-CD56 trended towards worse patient outcomes, only CD56 expression was found to be significant. A limitation of our study is our cohort size; a larger patient cohort would have strengthened our statistical output. Our evaluation of TIL CD56 expression found that the majority of tumors with CD56+ TILs were also positive for polySia and the presence of these TILs was an extremely favorable prognostic indicator. However, tumors with polySia+ TILs that were CD56-negative were significantly associated poor patient outcome. This demonstrates that there are different populations of polySia-expressing lymphocytes in the tumor microenvironment each with distinct prognostic significance. Likely, this explain why when we performed a general assessment of polySia expression we found high polySia TIL expression associated with poor outcomes and moderate/low expression associated with good outcomes. Separating out the polySia+, CD56+ TIL-expressing tumors from the polySia+, CD56− TIL-expressing tumors revealed polySia as a good prognostic indicator in the context of CD56+ TIL.

The role of CD56+, polySia+ lymphocytes in cancer is relatively uncharacterized. Given our results, CD56+, polySia+ TILs may represent an important immune subset with potential implications for improving patient outcomes. CD56 is most often associated with NK cells (part of the innate immune cell repertoire) but it is most unquestionably not limited to NK cells and has been shown to be expressed by some T cell subsets including gamma delta (γδ) T cells and activated CD8+ T cells^25^. The cytotoxic function of NK cells and cytotoxic T cells has an important role in the elimination of tumor cells but the role of CD56 and polySia in immune function is not well characterized. Some studies have shown that CD56+ γδ T cells display potent antitumor activity and CD56+ T cells display enhanced cytotoxicity compared to CD56− T cells^26,27^. NK cells upregulate their CD56 expression upon activation, while in an immunosuppressive microenvironment NK cells lose their CD56 expression and cytotoxic abilities, thus CD56 can be used as an NK activation marker^28^. In humans, and in the context of lymphocytes, polySia has been documented to occur on NK and CD3+ T cells with CD56 being a carrier protein and, in line with our work, these studies also found ST8Sia4 to be the sialyltransferase responsible for polySia synthesis^16,17,29^. In addition, for NK cells, CD56 and polySia expression were reported to increase following activation with IL-2 suggesting polySia levels are regulated by NK cell activation state. While CD56 and polySia expression clearly fluctuate with activation state, whether or not they have a direct role in immune function is not well described. A small number of studies support an active role for CD56 in immune function and it has been described that CD56 homophilic interactions between immune cells and cancer cells can mediate tumor cell killing^30,31^. How polySia may influence CD56-mediated tumor cell killing is relatively uncharacterized, but given the well described role of polySia in regulating a diverse array of cell and receptor interactions it likely has a role in mediating immune cell function. Identifying the immune subsets expressing polySia and determining the molecular underpinnings of polySia and CD56 function in the context of cancer will be an important next step.

## Materials and methods

### Antibodies and reagents

Anti-polysialic acid antibody [Clone 735] was purchased through Absolute Antibody Ltd. (Cleveland, UK: Cat. No. Ab00240-2.0; RRID:AB_2619682). Anti-NCAM1/CD56 antibody was purchased through Santa Cruz Biotechnology (Dallas, TX: Cat. No. sc-7326, clone123c3; RRID: AB_627127), anti-CD3 and Estrogen Receptor from Abcam (Toronto, ON: Cat. No. ab5690; RRID:AB_305055; ab16669, RRID:AB_443425; ab32063, RRID:AB_732249). All reagents for in situ hybridization using the RNAscope^®^ 2.5 HD Duplex Assay were purchased through Advanced Cell Diagnostics (Hayward, CA). RNAscope^®^ Probes are as follows: Hs-ST8SIA2 (Cat. No. 540411), Hs-and ST8SIA4-C2 (Cat. No. 540401-C2). All reagents for immunocytochemistry were purchased through Vector Laboratories Inc (Burlington, ON): Vectastain ABC-AP Kit (Cat. No. AK-5001), anti-Rabbit IgG Antibody (H + L), Peroxidase (Cat. No. PI-1000), and anti-Mouse IgG Antibody (H + L), Peroxidase (Cat. No. PI-2000).

### Human tissue and ethics

Breast cancer tissue sections and microarrays were obtained through the Ontario Tumor Bank and from US Biomax (Rockville, MD, USA). Biological materials, provided by the Ontario Tumor Bank (OTB), is supported by the Ontario Institute for Cancer Research through funding provided by the Government of Ontario. Tissue microarrays from US Biomax: Normal adjacent/Cancer adjacent (BRN801c), Staging (TMA #1 and #2: BR10010/BR20819), Outcomes (TMA #3 HBRe139Su01 and TMA#4 HBRe140Su07). Clinical information was provided from US Biomax and the Ontario Tumor Bank (OTB) on patient age, time to recurrence (OTB only), overall survival status, and tumor characteristics (N Stage, T stage, Grade). Molecular marker (ER, PR, HER2) expression, as evaluated by a pathologist, was provided for all OTB samples. For OTB cases where HER2 expression was equivocal, fluorescence in situ hybridization (FISH) was performed to classify tumors as HER+ or HER2−. For TMA #1 and #2, pathologist scoring of molecular markers (ER, PR, and HER2) was provided on *n* = 123 of 144 cores. Molecular markers were not provided for cores when tissue was missing or in cases where only fibrofatty tissue and blood vessels were present (i.e. no tumor cells). No molecular markers were provided for outcomes TMA #3. For outcomes TMA #4, ER, PR, HER2, FISH, and Ki67 expression was provided for each tissue core. See Supplemental Fig. 1 for additional details on patient cohorts. Stratified randomization method was applied to the clinical samples and included only individuals diagnosed with Invasive Ductal Carcinoma; tissue cores from individuals diagnosed with Invasive Lobular Carcinoma (Staging, *n* = 6 and Outcomes, *n* = 9), Lobular carcinoma in situ (Staging, *n* = 2 and Outcomes, *n* = 0), Medullary carcinoma (Staging, *n* = 6 and Outcomes, *n* = 0) (*n* = 6), Glycogen-rich clear cell carcinoma (Staging, *n* = 2 and Outcomes, *n* = 0), mucinous carcinoma (Staging, *n* = 0 and Outcomes *n* = 6), and intraductal carcinoma (Staging, *n* = 0 and Outcomes *n* = 1) were not included in the analysis. Study inclusion was female breast cancer patients, no male breast cancer patient samples were used. The study is compliant with all relevant ethical regulations on the use of human tissue and study approval was obtained from the Institutional Ethics Review Board of UBC (IRB#H17-01442).

### Immunohistochemistry and digital image analysis and scoring

Human paraffin-embedded breast cancer tissue microarray sections (TMAs) were incubated overnight at 37 °C, followed by deparaffinized with xylene and rehydration using an ethanol gradient followed by a 1Xphosphate-buffered saline (PBS), pH 7.4, wash. For CD56 and CD3 immunostaining heat-induced antigen retrieval was performed at 95 °C for 40 min in a 10 mmol/L citrate buffer (pH 6). After heating, slides were cooled to room temperature and washed in 1XPBS. No antigen retrieval was performed for polySia immunostaining. Slides were then incubated with 0.1% Triton X-100 in PBS for 10 min followed by a 20 min incubation in 10% serum from the host of each secondary. TMAs were washed with PBS and incubated with primary antibody (1:500 for polysialic acid, 1:100 for CD56, and 1:200 for CD3 [ab5690]) for 1 h (polysialic acid and CD56) or 0.5 h (CD3) at room temperature. Excess antibodies were removed by washing three times with 1XPBS. TMAs were incubated for 1 h at room temperature with a biotinylated horse anti-mouse IgG or goat anti-rabbit IgG followed by horseradish peroxidase (HRP) streptavidin for 30 min at room temperature and the stained with DAB (3-3’-Diaminobenzidine) (Vector Laboratories). Slides were counterstained with Harris hematoxylin, dehydrated and mounted with Vecta mount medium (Vector Laboratories).

For chromogenic dual staining of ER and polySia, slides were deparaffinized and heated in EDTA antigen retrieval buffer (pH 8) for 20 min, incubated with 0.1% Triton X-100 solution, followed by a 20 min incubated with BLOXALL^®^ blocking solution. Slides were incubated with anti-estrogen receptor alpha antibody (ab32063) for 1 h at room temperature, followed by incubation with a secondary antibody (biotinylated goat anti-rabbit IgG), then ABC-alkaline phosphatase reagents, and finally ImmPACT Vector Red substrate. Following this, slides were incubated with anti-polySia antibody for 1 h at RT, incubated with secondary antibody followed by HIGHDEF^®^ yellow IHC chromogen. Slides were counterstained with Hematoxylin and mounted with Vactamount medium.

For CD3 and polySia dual immunofluorescent staining, FFPE slides were deparaffinized, dehydrated and heated in EDTA antigen retrieval buffer (pH 8) for 20 min. After permeabilization and serum blocking, slides were incubated with anti-CD3 antibody (ab16669) at 1:100 and Alexa 488-conjugated polysialic acid antibody (ab00240-2.0) at 1:100 for 1 h at RT. Slides were washed with PBS, incubated with Sudan Black solution for 10 min, followed by incubation with Alexa 594-conjugated Rabbit IgG secondary antibody. Nuclei were stained using Hoechst. The slides were mounted with Dako Fluorescent Mounting Medium.

For CD56 and polySia dual immunofluorescent staining, FFPE slides were deparaffinized, dehydrated, permeabilized, and blocked with 10% serum. Slides were incubated with rabbit anti-polysialic acid at 1:125 (ab00230-2.0) for 1 h at room temperature. Slides were washed with PBS 3 × 5 min followed by incubation with PE-NCAM1 (Abcam. Cat. No. ab18277) at 1:50 and anti-rabbit Alexa Flour 488 (Invitrogen, Cat. No. A11008) at 1:3000 for 1 h at RT. Slides were washed with PBS and autofluorescence quenched using Vector True VIEW Autofluorescence Quenching Kit (Vector Laboratories, CA, USA) for 5 min. Cell nuclei were stained with Hoechst 33342 Solution (Thermo Fisher Scientific, Cat. No. 62249) at 1:5000 dilution for 10 min. Slides were mounted with fluorescent mounting medium (Dako, Cat. No. CS70330-2) and images were captured by Zeiss Axio Observer microscope (Carl Zeiss, Germany) at ×20 magnification.

Stained TMA slides were digitalized with the SL801 autoloader and Leica SCN400 scanning system (Leica Microsystems; Concord, Ontario, Canada) at magnification equivalent to ×40. Pathologist evaluation of tumor cells and tumor-infiltrating lymphocytes was performed using the Aperio ImageScope IHC menu (Leica Biosystems) where areas of interest (tumor or TILs) were selected and evaluated using the positive pixel Count Algorithm for each marker. The digital score-based algorithm reports a value between 0 and 1 based on the intensity and percentage in the given area; this value is reported as the *H*-Score. All scoring was performed blinded to clinical data.

### In-situ hybridization and analysis using ZEN Intellesis software

The RNAscope® chromogenic assay (Advanced Cell Diagnostics, Hayward, CA) was used to detect mRNA molecules which are visualized as punctate dots. ST8Sia2 and ST8Sia4 RNAscope probes were designed and provided by Advanced Cell Diagnostics, Hayward, CA. RNAscope probes were used based on manufactures instructions. RNAscope^®^ Probes used:-Hs-ST8SIA4-C2 Cat# 540401-C2, Hs-ST8SIA2 Cat# 540411. Briefly, TMAs were baked at 60 °C for 1 h, deparaffinized using Xylene and 100% ethanol, and air-dried before pretreatments. Slides were then incubated in retrieval buffer at boiling temperature (100 °C) for 15 min, rinsed in deionized water, EtoH and immediately treated with protease at 40 °C for 30 min in a HybEZ-hybridization oven (Advanced Cell Diagnostics, Hayward, CA). TMAs were hybridized using both ST8SIA2 and ST8SIA4 probes. Control slides were hybridized with positive control probe Hs PPIB - C1 (PN 321641) and 2 Plex-Negative control probe DapB-C1/DapB-C2 (PN 320751). All probes were incubated at 40 °C for 2 h in the HybEZ oven. Slides were washed twice with 1X wash buffer 2, 2 min each at room temperature, and signals were amplified through a 10-step incubation using RNAscope 2.5 HD Reagents Duplex Detection Kit. (ACD, Cat. No. 322435).

Each TMA core was scanned using a Zeiss Axio Observer microscope microscope slide scanner (Carl Zeiss, Germany) at ×20 magnification. Image segmentation and analysis was performed using ZEN Intellesis deep-machine-learning program software (ZEN Intellesis, ZEN 2.6 Blue edition software, Zeiss, Germany). ZEN Intellesis software was used to train and develop a program which could identify nuclei and differentiate between ST8SIA2 and ST8SIA4, or CD56 based on pixel color. The program was then applied to each TMA and image segmentation was performed. Tumor and stromal areas for each tissue section were manually selected and each area was segmented. The digital output reports the total number of chromogenic RNA punctate for each color and total number of nuclei. The results are reported as the total counts of RNA punctate divided by the total nuclei counts. This represents the average mRNA expression per cell. All scoring was performed blinded to clinical data.

### Statistics

Data was handled in Excel (Microsoft Excel; RRID:SCR_016137) and analyzed using GraphPad Prism 8.0.1 (GraphPad Prism, RRID:SCR_002798). Column analysis for two data sets was analyzed using a two-tailed unpaired *t*-test. Welch’s correction was applied to any data sets with significant variances (*F* test to compare variances). Multiple comparisons between greater than two groups used an ordinary one-way ANOVA Tukey’s multiple comparisons test. All data sets were analyzed for normality using the Shapiro–Wilk test. If normality failed, data was transformed using either *Y* = sqrt(*Y*) or *Y* = logit(*Y*). Transformed data was then analyzed for normality using the Shapiro–Wilk test; if normality failed again, data was analyzed using non-parametric Mann–Whitney *t*-test or Kruskal–Wallis ANOVA. *p < 0.05, **p < 0.01, ***p < 0.001, ****p < 0.0001.

## Supplementary information

Supplemental File

## Data Availability

All datasets generated during and/or analyzed during the current study are available from the corresponding author on reasonable request.
