# Supplementary material for: The different prognostic significance of polysialic acid and CD56 expression in tumor cells and lymphocytes identified in breast cancer
Source: NPJ Breast Cancer. 2022 Jul 2;8:78. doi: 10.1038/s41523-022-00442-w (PMC9250520; doi:10.1038/s41523-022-00442-w)
Supplement: Supplementary file 1 — Supplemental File [file 41523_2022_442_MOESM1_ESM.pdf]

SUPPLEMENTARY DATA

The Different Prognostic Significance of Polysialic Acid and CD56 Expression in Tumor Cells and Lymphocytes Identified in Breast Cancer

Sepideh Soukhtehzari,[1] *Richard B. Berish,*[1] *Ladan Fazli,* MD[2] *Peter H. Watson,* MD[3] *and Karla C. Williams,* PhD[1*]

Supplemental Figure 1

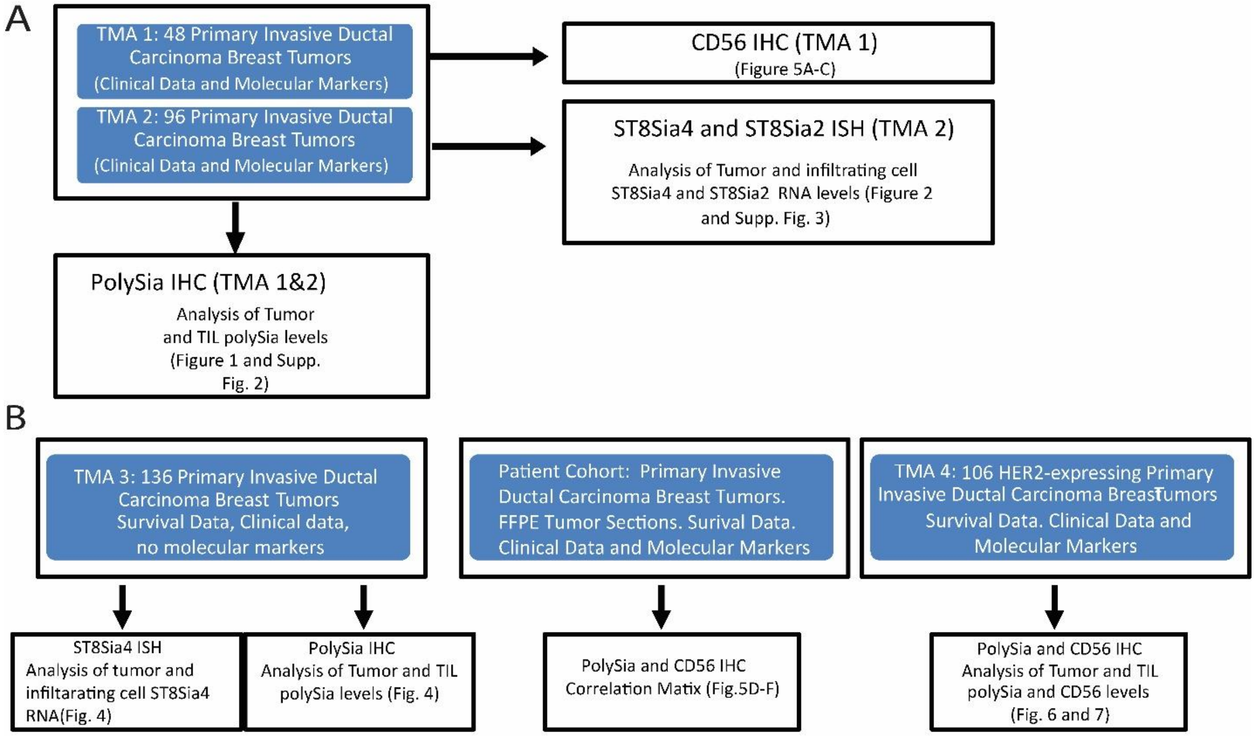

C

Subtype	ER	PR	HER2	ki67
Luminal A	ER positive and/or PR positive		Negative	<14%
Luminal B	ER positive and/or PR positive		Positive, or ki67>14%	
HER2+	Negative	Negative	Positive	Any ki67
TNBC	Negative	Negative	Negative	Any ki67

D

TMA #	LumA	LumB	HER2	TNBC
#1: IHC Expression Data for ER, PR HER2	20	1	4	22
#2: IHC Expression Data for ER, PR HER2	40	8	8	20
#3: No Expression Data	n/a	n/a	n/a	n/a
#4: IHC Expression Data for ER, PR, HER2, and Ki67. FISH data for HER2	28	35	25	18

Tissue Sections (n=33)	LumA	LumB	HER2	TNBC
IHC Expression Data for ER, PR, HER2 FISH data for IHC HER2 Equivocal Cases	11	3	6	13

Supplementary Figure 1: Breakdown of clinical tissue samples. (A) Diagram showing the tissue microarrays used in the analysis of polySia and ST8Sia4. **(B)** Diagram of tissues with clinical outcomes data reporting overall survival and their corresponding use in the study. **(C)** Molecular subtype classification based on receptor expression. HER2 status was defined as negative when the IHC membrane score was 0 or 1+. In cases with equivocal HER2 expression (2+), HER2 FISH data was used to determine positivity. HER2 FISH data was provided for Outcomes TMA #4 and the patient cohort (tissue sections/ OTB samples). For TMA #1 and #2, in the absence of FISH data, HER2 equivocal cases were classified as HER2 negative. Outcomes TMA #4 was the only TMA with Ki67 expression data and this was used in molecular subtype classification. **(D)** An overview of each TMA/cohort, the molecular markers provided, and the number of cases per cohort.

Supplemental Figure 2

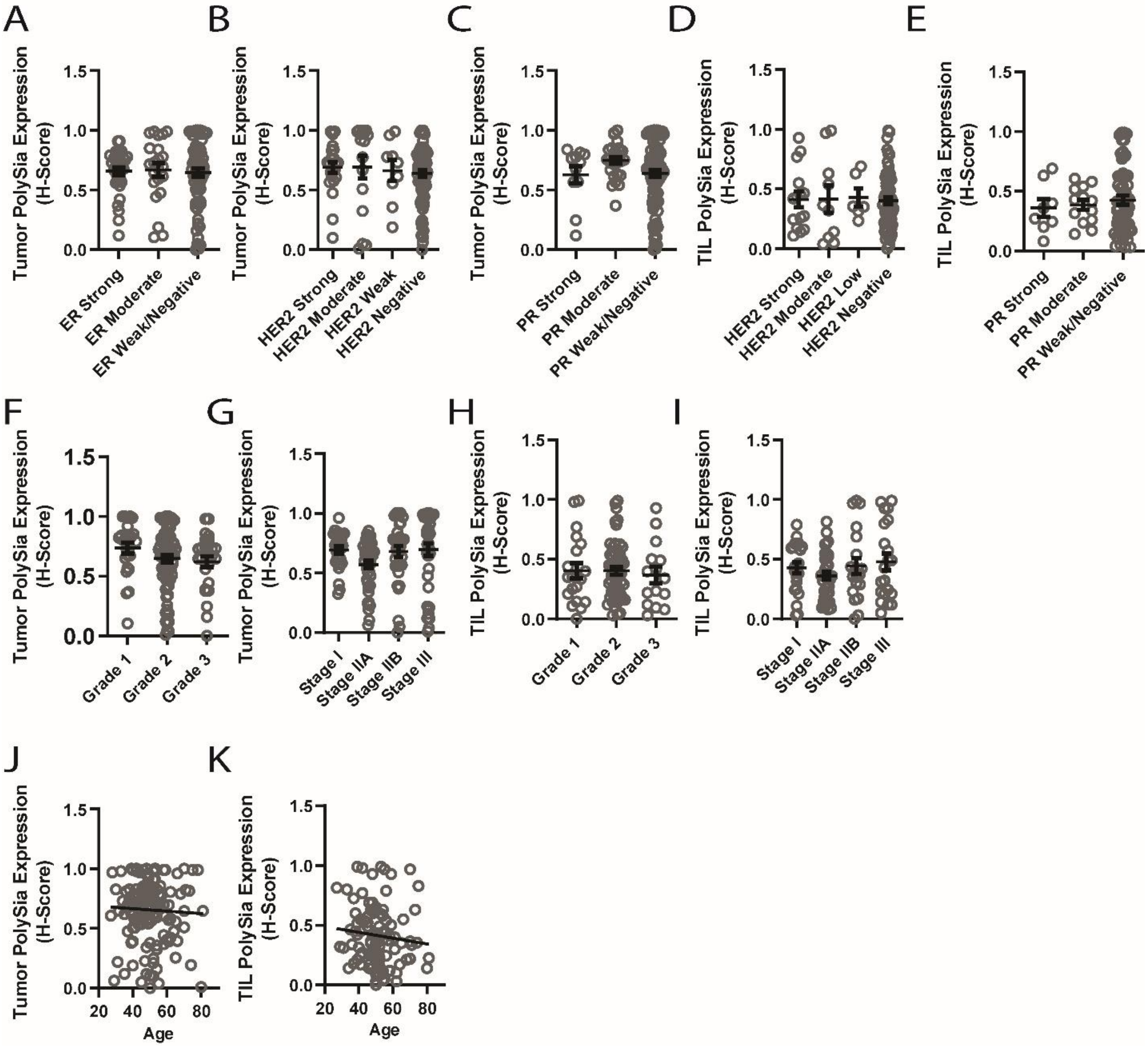

Supplementary Figure 2: Assessment of polySia TIL and tumor cell expression based on clinical parameters. **(A-C)** Tumor cell polySia expression categorized by estrogen receptor (ER) **(A)**, HER2 **(B)**, and progesterone receptor (PR) **(C)** status. **(D and E)** Tumor infiltrating lymphocyte (TIL) cell polySia expression categorized by HER2 **(D)** and PR **(E)** receptor status.**(F-G and J)** Tumor cell polySia expression based on overall tumor grade **(F)**, stage **(G)**, and patient age at time of diagnosis **(J)**. Tumor infiltrating (TIL) cell polySia expression based on overall tumor grade **(F)**, stage **(G)**, and patient age at time of diagnosis **(K)**.

Supplemental Figure 3

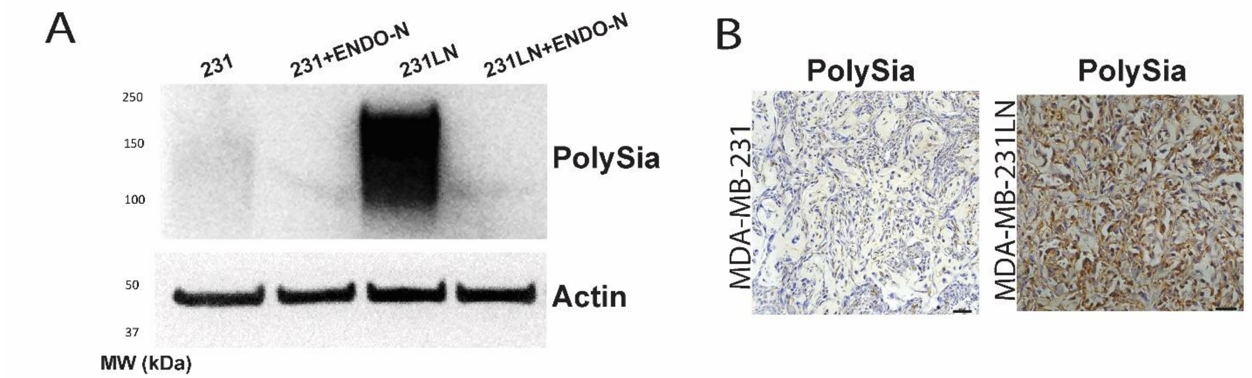

Supplementary Figure 3: Validation of polySia antibody binding specificity. **(A)** Expression of polySia in breast cancer cell line MDA-MB-231 (231) and metastatic derivative MDA-MB-231-luc-D3H2LN (231LN). PolySia expression is shown in the 231LN cell line and further validated by cleavage using the polySia specific, and selective, enzyme endoneuraminidase N (ENDO-N). **(B)** Immunohistochemical staining of tumors generated using 231 and 23LN cell lines grown on the chick embryo chorioallantoic membrane (CAM). Tumor sections immunostained for polySia show expression of polySia only in the 231LN tumors. Scale bar=50μm.

Supplemental Figure 4

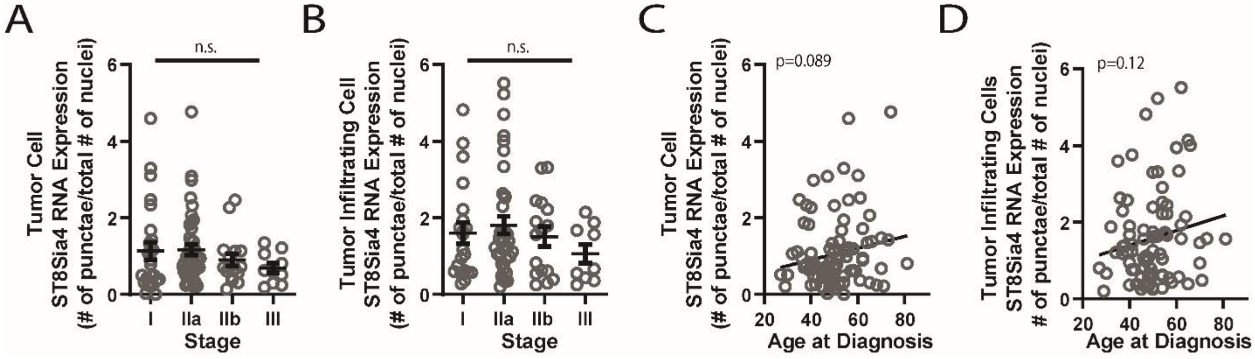

Supplementary Figure 4: ST8Sia4 expression in tumor cells and infiltrating stromal cells categorized by stage and age. Tumor cell ST8Sia4 RNA expression based on stage **(A)** and age **(C)**. **(B and D)** Tumor infiltrating stromal cell ST8Sia4 RNA expression based on stage **(B)** and age **(D)**.

Supplemental Figure 5

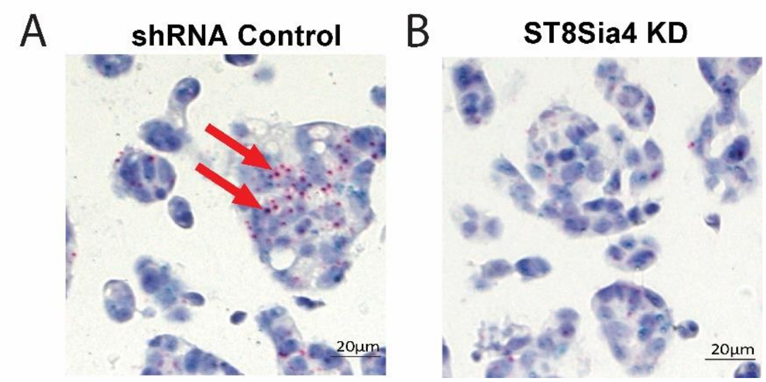

Supplementary Figure 5: ST8Sia4 RNA probe validation. Stable shRNA control and shRNA against ST8Sia4 (ST8Sia4 KD) cell lines were generated using MCF7 cells. Tumor xenografts were generated using the chick embryo model. Tumors were fixed, sectioned, and in situ hybridization was performed for ST8Sia4 (red) and ST8Sia2 (green). **(A)** ST8Sia4 RNA punctae are readily observed by ISH on control tumors (red arrows); a few ST8Sia2 punctae are also observed (green punctae). **(B)** ST8Sia4 RNA punctae by ISH on knockdown tumors showing that only a few punctae are observed and no change in RNA punctae is noted for ST8Sia2 (green punctae). Representative images are shown. Scale bar=20µm

Supplemental Figure 6

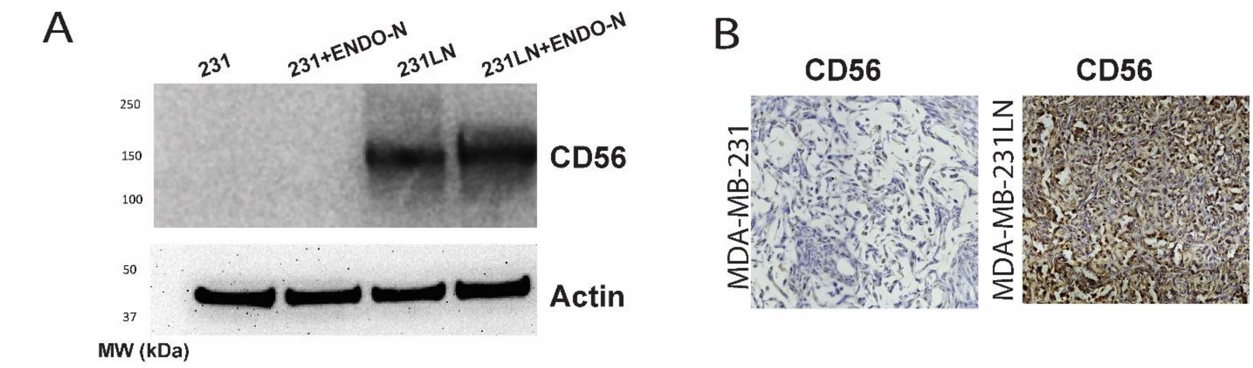

Supplementary Figure 6: Validation of CD56 antibody binding specificity. **(A)** Expression of CD56 in breast cancer cell line MDA-MB-231 (231) and metastatic derivative MDA-MB-231-luc-D3H2LN (231LN). CD56 expression is shown in the 231LN cell line as a single prominent band with a smear above. Cleavage of polySia chains using the polySia specific, and selective, enzyme endoneuraminidase N (ENDO-N) reduced this smear and a thicker CD56 band results. **(B)** Immunohistochemical staining of tumors generated using 231 and 23LN cell lines grown on the chick embryo chorioallantoic membrane. Tumor sections immunostained for polySia show expression of polySia only in the 231LN tumors. Scale bar=50µm.
